# Single-day accelerated TMS with D-cycloserine augmentation for depression: A placebo-controlled trial

**DOI:** 10.21203/rs.3.rs-7980692/v1

**Published:** 2025-12-11

**Authors:** Prem Ganesh, Jamie Kweon, Julia Tom, Hakjoo Kim, Giuseppe Varone, Alexander McGirr, Joshua Brown

**Affiliations:** McLean Hospital; McLean Hospital; McLean Hospital; University of Calgary; McLean Hospital

## Abstract

Accelerated transcranial magnetic stimulation (TMS) protocols have the potential to rapidly treat depression, yet the synaptic mechanisms underlying these intensive interventions remain poorly understood. d-cycloserine (DCS), a partial NMDAR agonist, enhances TMS-induced corticomotor plasticity and conventional daily TMS outcomes, but its effects on accelerated protocols are unknown. We conducted a double-blind placebo-controlled trial examining whether DCS could enhance single-day accelerated intermittent theta-burst stimulation (iTBS) in 30 participants with major depressive disorder. Participants received either 250 mg DCS or placebo the night before undergoing 10 iTBS treatments (1 800 pulses/treatment) delivered hourly to the left dorsolateral prefrontal cortex. Motor-evoked potentials (MEPs) were recorded before and after each treatment to assess corticomotor excitability, while depression severity was measured at baseline and one-week post-treatment using PHQ-9 and QIDS-SR16 scales. Normalized MEP amplitudes were analyzed using separate generalized linear mixed models for pre- and post-iTBS measurements, revealing a significant Group × Treatment interaction in only the pre-iTBS data (χ^2^ = 4.19, p = .041), with placebo showing increasing trajectories and d-cycloserine remaining stable. However, post-iTBS measurements showed no Group × Treatment interaction (χ^2^ = 0.92, p = .338), indicating no differential plasticity responses. Clinical outcomes showed improvement over time on QIDS-SR16 (p = .047) but not PHQ-9 (p = .206), with no between-group differences on either scale (PHQ-9: p = .112; QIDS: p = .286). These findings suggest that high-intensity accelerated protocols may reach a plasticity ceiling that occludes further synaptic enhancement with NMDAR agonism, highlighting the importance of parameter optimization for pharmacologically-augmented accelerated TMS.

## INTRODUCTION

Rapid and robust clinical improvements, as the ultimate aim of transcranial magnetic stimulation (TMS) research, are most likely to be attained through its underlying mechanisms of action^1^ and optimization of TMS parameter space^2^. Recent advances in noninvasive brain stimulation protocols and pharmacological augmentation are steps towards bringing this objective within reach^3–5^.

Two major innovations in the field of TMS have taken notable steps toward this goal. First, recent accelerated protocols, which deliver more than one treatment per day, have yielded response rates that would otherwise require 10 weeks with daily TMS^6–8^ in only 5 days (Cole et al., 2022). Second, by leveraging the putative mechanism of repetitive TMS, namely long-term potentiation (LTP) with d-cycloserine (DCS), a partial agonist of the *N*-methyl-d-aspartate receptor (NMDAR), traditional daily TMS protocols have seen large enhancements in clinical efficacy for major depressive disorder (MDD) and obsessive-compulsive disorder (OCD) with large effect sizes^3, 9^. Furthermore, a recent open-label cohort describes the combination of these strategies by pairing adjuvants with the potential to enhance LTP-like effects with as many as 20 treatments in a single day.

Despite these advances, important questions remain unanswered. First, how do accelerated protocols therapeutically change the brain at the rate-limiting synaptic-level? While single-session work suggests that TMS may effect change through LTP mechanisms in animals and LTP-like effects humans^10–14^, there are several dimensions of the accelerated parameter space that are not fully elucidated nor their mechanistic requirements known. These include, but are not limited to, the stimulation protocol itself as varying train lengths and number of pulses have different or opposite effects, the number of stimulations before occluding synaptic level changes or destabilizing/rescaling recent adaptation, the inter-stimulus interval required for metaplastic (the plasticity of plasticity) effects. Furthermore, these are likely factorial and the specific combinations having different circuit level effects, and their NMDA receptor dependence remains to be determined. To date, a single study using a 600 pulse intermittent theta-burst protocol and D-Cycloserine augmentation provides physiological data to suggest that two repeated treatments with approximately 60 minute inter-treatment interval increases corticospinal excitability over stimulation with a placebo^15^. Yet in clinic the boundaries are being rapidly tested with up to 20*600 pulse iTBS treatments per day being delivered with D-Cycloserine with only 30 minute inter-treatment intervals^16^. How pharmacological augmentation intersects with the supraphysiologic 1 800-pulse iTBS protocol^17^ commonly used in accelerated treatment protocols^9^ remains unknown.

Here, we conducted a randomized placebo-controlled trial testing the clinical effects of pairing D-Cycloserine augmentation with 10*1 800 pulse iTBS accelerated treatment protocols that are increasingly in clinical use. We do so for a single day of treatment, and simultaneously sample cortical excitability across the protocol, as well as critical biological confounds including drug levels and genetic variation in Brain Derived Neurotrophic Factor (BDNF). The a priori hypothesis is that DCS would enhance TMS-induced clinical outcomes and neuroplasticity and, however we recognize that the supraphysiological nature of the 1 800 pulse iTBS protocol could result in occlusion of plasticity and metaplasticity with NMDA receptor agonism.

## MATERIALS AND METHODS

### Participants

**Figure 1:**
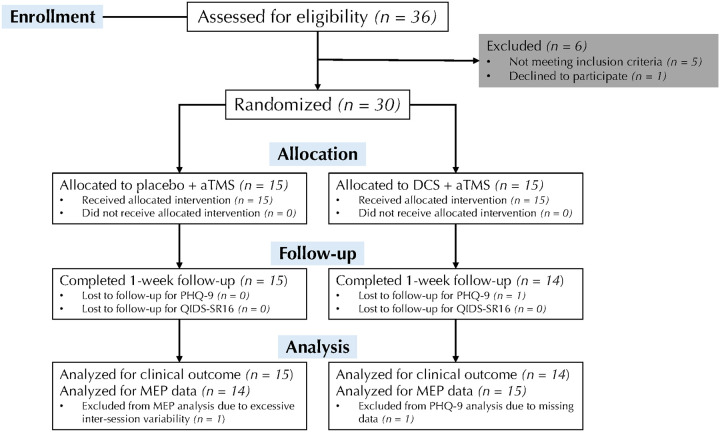
CONSORT diagram.

In this double-blind parallel arm study, we enrolled 30 subjects (19 female) aged 18–69 years (average 38.5 ± 16.0 years) with major depressive disorder (MDD) from the McLean Hospital Outpatient TMS Clinic (Belmont, MA, United States). Diagnosis for each subject was determined by a board-certified psychiatrist. All subjects provided informed consent. Full demographic information can be found in Table 1. All study components were approved by the Mass General Brigham Institutional Review Board. Exclusion criteria were known DCS allergy, TMS contraindications such as implanted metal in the skull and elevated seizure risk, and magnetic resonance imaging (MRI) contraindications. Full enrollment and participation information can be found in Figure 1.

### Magnetic resonance imaging (MRI) visit

All participants underwent MRI at the McLean Imaging Center, Belmont, MA. The imaging sequence included a T1-weighted MPRAGE anatomical scan (TR = 2.3s, TE = 2.32 ms, TI = 0.9s, flip angle = 8°, 0.9mm isotropic voxels, 256 × 256 matrix, acceleration factor = 2) which was uploaded to the neuronavigation software.

Following the scan, the motor hotspot of the right abductor pollicis brevis (APB) muscle was determined via mapping of the left primary motor cortex.

Participants received a single dose of either 250 mg DCS or an identical microcrystalline cellulose capsule and were instructed to take the capsule the night before the TMS study day. Thus, participants ingested the capsule ~10 hours prior to treatment 1, representing 1–2 drug half-lives^18^. Accordingly, even if serum concentrations achieved NMDA receptor antagonist concentrations due to its properties as a partial agonist, the serum concentration for the duration of the experiment would remain within the NMDA receptor agonist range. This differs from our previous approach of 100 mg ingested 2 hours before the study^19^, which is difficult to integrate with long treatment days and clinical samples have well identified adherence challenges with timed ingestions^4^. Participants were randomized 1:1 using permuted block randomization (block size = 4) generated by Sealed Envelope (www.sealedenvelope.com). The computer-generated allocation sequence was prepared by the McLean Research Pharmacy prior to enrollment. Treatment assignments were concealed until the point of allocation. All investigators, participants, and study staff remained blinded to the treatment assignment throughout the study and analysis.

### Transcranial magnetic stimulation visit

Resting motor threshold (rMT) was calculated using Nexstim MT algorithm^20^ to establish stimulation intensities for treatment and research measures. All TMS was MRI-guided, and all pulses were manually kept within 0.5 mm of target.

iTBS (50-Hz triplet bursts within a 5-Hz carrier frequency, 2 second on, 8 seconds off, 1 800 pulses) was administered every hour at 100% of the rMT maximum real-time electric-field (e-field) to the left dorsolateral prefrontal cortex (dlPFC) using anatomical-based targeting^21^ with a Nexstim NBS 6 TMS device (Helsinki, Finland). The rationale for this intensity dosing approach aimed to remove the variability that occurs with estimated intensity based simply on a 20% percent increase from motor threshold but takes coil to cortex distance into account at the precise location of stimulation^22, 23^.

### Motor-evoked potentials

Motor-evoked potentials (MEPs) were recorded from the right APB muscle with disposable pre-gelled electromyography (EMG) electrodes. EMG signals were sampled and peak-to-peak amplitudes calculated within Nexstim NBR software (version 1.0, Nexstim Plc, Helsinki, Finland). 20 MEPs were collected immediately before and after all 10 aTMS treatments for a total of 20 timepoints. MEPs were evoked by single pulses sent to the motor hotspot with a stimulator intensity matched to 120% of the rMT e-field. Pulses were jittered 4–7 seconds apart.

### Blood samples

d-cycloserine serum levels were found to be strongly associated with clinical outcomes in daily TMS protocols (DeMayo et al., 2025). Accordingly, serum samples were obtained twice for each subject: before treatment #1 (AM, before midday) and before treatment #10 (PM, after midday). We collected two, 6 mL BD Vacutainer Serum Blood Collection Tubes using standard phlebotomy procedure. Separated serum was aliquoted and frozen samples were sent to Advanced Diagnostic Laboratories at National Jewish Health via Quest Diagnostics for DCS serum level analysis using Gas Chromatography/Mass Spectrophotometry (GC/MS). Blood could not be obtained at the following 6 timepoints due to unsuccessful draw: Subject 1 PM, subject 3 PM, subject 8 PM, subject 12 AM and PM, subject 16 PM, and subject 24 PM.

### Saliva samples

One saliva sample was collected from each subject for genetic testing prior to the last treatment (i.e., treatment #10). Frozen samples were sent to Salimetrics for DNA extraction and SNP analysis (rs6265) for brain-derived neurotrophic factor (BDNF), which we considered as a covariate for TMS-induced plasticity^24^. Samples were not collected from the first and second participants, and therefore available for *n* = 28 participants.

### Clinical scales

On the day of the aTMS visit, the 16-item Quick Inventory of Depressive Symptomology Self-Report (QIDS-SR16) and Patient Health Questionnaire-9 (PHQ-9) were administered^25, 26^. Clinical scales were repeated one week following the aTMS day. One subject did not complete the PHQ-9 follow-up survey.

**Figure 2:**
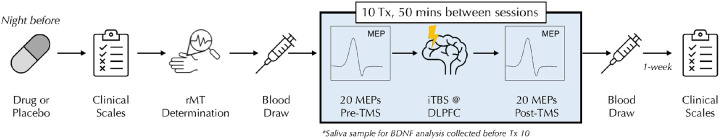
Study design. DLPFC = dorsolateral prefrontal cortex; MEP = motor-evoked potential; rMT = resting motor threshold; iTBS = intermittent theta burst stimulation; Tx = treatment; BDNF = Brain-derived Neurotrophic Factor.

Fig. 2 illustrates the study design.

### Data analysis

To evaluate between-subjects effects of drug and within-subjects effects of time on MEP amplitudes, MEP values were first normalized to each subject’s baseline (pre-treatment 1) and averaged within each subject and timepoint to ensure statistical independence. We used generalized linear mixed-effects models (GLMMs) with a Gamma distribution and log link function to account for the positive, right-skewed nature of normalized MEP data. To isolate baseline stability from plasticity responses, analyses were conducted separately on pre-iTBS and post-iTBS measurements. Each model included Group (PBO vs. DCS) and Treatment (1–10, standardized) as fixed effects with their interaction term, and random intercepts for both subject and subject-by-treatment. Statistical significance was assessed using Type II Wald chi-square tests. Model assumptions were verified through visual inspection of residual plots and Shapiro-Wilk tests for normality of residuals. The pre-iTBS model tests whether corticospinal excitability trajectories differed between groups across the experimental day; note that pre-iTBS measurements (except at treatment 1) occur 50 minutes following the prior session’s stimulation and may reflect both baseline stability and delayed facilitation effects. The post-iTBS model tests whether groups showed differential plasticity responses immediately following stimulation.

Clinical outcomes were analyzed using two-way analysis of variance (ANOVA) with Group (DCS vs. PBO) and Time (baseline vs. 1-week post-TMS) as factors. Normality assumptions were assessed using the Shapiro-Wilk test for each group-time combination. For PHQ-9 data, normality was satisfied across all conditions (all p > 0.15), and homogeneity of variance was confirmed using Levene’s test (p = .249). For QIDS data, normality was similarly satisfied across conditions (all p > 0.07) with homogeneous variances (Levene’s p = .211). Given that parametric assumptions were met, two-way ANOVA was conducted to test main effects of Group, Time, and their interaction.

To examine potential demographic predictors of treatment response, age was correlated with percent improvement scores using Pearson and Spearman correlations, while sex differences in treatment response were evaluated using Mann-Whitney U tests. No significant demographic associations were found (all p > 0.15).

All statistical analyses were done in R software (version 4.2, R Core Team, Vienna, Austria) and SPSS (version 28.0.0.0, IBM, Armonk, NY, United States). Visualizations were generated in Python (version 3.10) and GraphPad Prism (version 10.6.0, GraphPad Software, Boston, MA, United States).

## RESULTS

Thirty participants with MDD completed the study protocol (15 placebo (PBO), 15 DCS). Demographic and clinical characteristics are presented in Table 1. Groups were well-matched for age (PBO: 41.8 ± 15.4 years; DCS: 35.2 ± 16.4 years), with a predominantly female (60.0%) and White (96.7%) sample. Baseline depression severity was comparable between groups on both the QIDS-SR16 (PBO: 14.9 ± 1.30; DCS: 13.1 ± 1.07) and PHQ-9 (PBO: 15.9 ± 1.4; DCS: 13.3 ± 1.3) scales, indicating on average moderate-to-severe depression severity.

**Figure 3:**
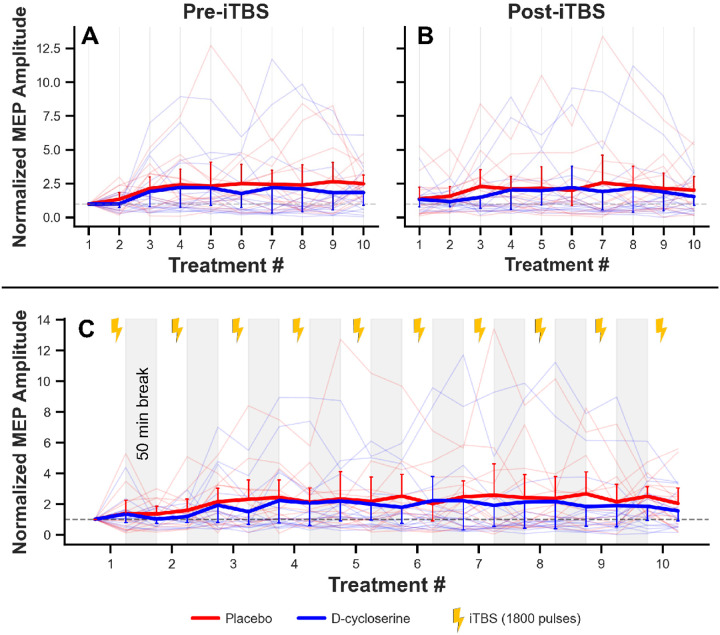
Normalized MEP trajectories across the experimental day. **(A) Pre-iTBS measurements, (B) post-iTBS measurements** across 10 treatment sessions, and **(C) All 20 timepoints**. MEP amplitudes normalized to each subject’s baseline (pre-treatment 1; dashed horizontal line at 1.0). Thick lines represent group means (red = placebo, blue = D-cycloserine), with one-sided directional error bars indicating SEM. Faded lines indicate individual subject trajectories. Light gray shading in panel C indicates 50-minute intervals between treatments. Yellow lightning icons represent 600-pulse iTBS protocol. Pre-iTBS measurements showed a significant Group × Treatment interaction (χ^2^ = 4.19, p = .041), with PBO showing an increasing trajectory and DCS showing no change. Post-iTBS measurements showed no Group × Treatment interaction (χ^2^ = 0.92, p = .338)

Baseline raw MEP amplitudes were 1.06 ± 0.17 mV across participants, with no significant differences between groups (PBO: 0.79 ± 0.21 mV; DCS: 1.34 ± 0.25 mV). Generalized linear mixed-effects modeling of normalized MEP amplitudes revealed no main effects of Group (χ^2^ = 2.16, p = .142) or Treatment (χ^2^ = 3.80, p = .051) in the pre-iTBS model, but a significant Group × Treatment interaction (χ^2^ = 4.19, p = .041). This interaction reflected opposing trajectories between groups: the DCS group exhibited minimal change across treatments (slope = −0.001), while the PBO group showed a gradual increase (slope = +0.150) (Fig. 3A). In the post-iTBS model, there were no main effects of Group (χ^2^ = 0.42, p = .518) or Treatment (χ^2^ = 1.21, p = .271), and no Group × Treatment interaction (χ^2^ = 0.92, p = .338). Both groups showed minimal slopes in the post-iTBS model (DCS: +0.006; PBO: +0.075), indicating no differential plasticity responses between groups (Fig. 3B).

**Figure 4:**
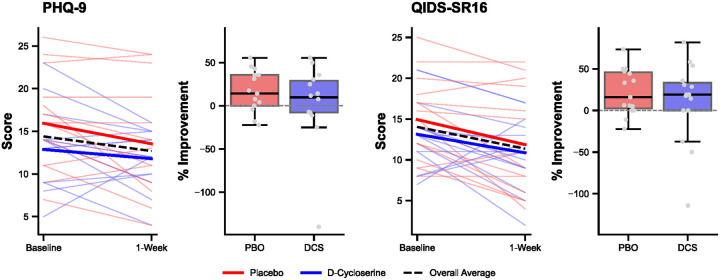
**Clinical improvement**, expressed as percent change from baseline to 1-week post-TMS, measured by two self-report scales: the Patient Health Questionnaire (PHQ-9, left) and the Quick Inventory of Depressive Symptomatology (QIDS-SR16, right). Line plots show raw score changes from baseline to 1-week follow up. Thick lines indicate group averages, faded lines indicate individual subject data. Box plots show the interquartile range (IQR; 25th to 75th percentile) and median. Whiskers represent 1.5 × IQR. Individual subject data are represented by gray dots. No significant differences between treatment groups were observed on either scale.

Two-way ANOVA of clinical outcomes revealed a significant main effect of Time (improvement over time) for QIDS-SR16 (F(1,56) = 4.21, p = .045) but not for PHQ-9 (F(1,54) = 2.10, p = .154) (Fig. 4). There were no significant main effects of Group (PHQ-9: F(1,54) = 2.62, p = .112; QIDS-SR16: F(1,56) = 1.16, p = .286) or significant Group × Time interactions (PHQ-9: F(1,54) = 0.11, p = .742; QIDS-SR16: F(1,56) = 0.09, p = .760), suggesting comparable treatment responses between DCS and placebo groups (Fig. 3). Response rates (≥ 50% improvement) were comparable between groups for both PHQ-9 (PBO: 6.7%; DCS: 14.3%) and QIDS-SR16 (PBO: 20.0%; DCS: 20.0%). Similarly, remission rates showed no between-group differences (PHQ-9 remission: PBO 6.7%, DCS 7.1%; QIDS-SR16 remission: PBO 20.0%, DCS 13.3%).

**Figure 5:**
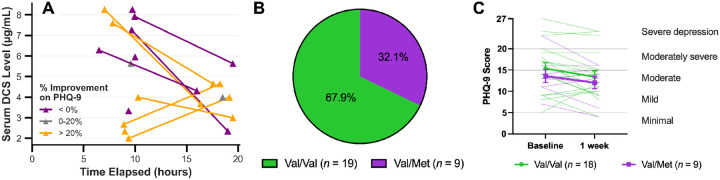
Plasticity covariates. **(A) Serum DCS levels**. Elapsed time in hours from time of drug administration (night before TMS). Each line represents on subject. Lines and dots are colored based on percentage improvement on PHQ-9. **(B) BDNF alleles**. Proportion of Val/Val (n = 19) and Val/Met (n = 9) carriers in our study population. (**C**) Clinical response by rs6265 variant as measured by PHQ-9 (not significantly different).

Serum DCS levels were analyzed from 13 of 15 subjects in the active group to test drug absorption and providing pharmacokinetic data across the treatment day (Fig. 5A). Differences in clinical outcomes were not associated with DCS levels at treatment onset.

BDNF rs6265 genotyping was completed for 28 participants, revealing 19 Val/Val carriers (67.9%) and 9 Val/Met carriers (32.1%) (Fig. 5B). No Met/Met carriers were identified in this sample. However, one participant in the Val/Val condition did not complete the post-TMS PHQ-9, so analyses were conducted on data from 27 participants. The Shapiro-Wilk test confirmed that baseline and post-TMS PHQ-9 scores were normally distributed within each group. A 2 (Condition: Val/Val, Val/Met) ×2 (Time: Baseline, 1 week) repeated measures ANOVA revealed a significant main effect of Time, F(1, 25) = 5.450, p = .028, partial *η*^2^= 0.179, but no significant effects of Condition (p = .455) or the Condition × Time interaction (p = .797) were found. These findings suggest that clinical response did not differ significantly between BDNF genotype groups as measured by PHQ-9 symptom improvement (Fig. 5C).

Demographic factors were examined as potential predictors of treatment response. Neither age (PHQ-9: r = 0.117, p = .547; QIDS: r = 0.115, p = .544) nor sex (PHQ-9: p = .156; QIDS: p = .280) were significantly associated with percent improvement on either scale when combining across treatment groups.

## DISCUSSION

In this double-blind placebo-controlled trial testing pharmacological enhancement of a single-day accelerated iTBS protocol, we hypothesized that D-cycloserine would enhance both neurophysiological markers of plasticity and clinical outcomes. Contrary to this hypothesis, analysis of motor-evoked potentials revealed no evidence that D-cycloserine modulated plasticity responses to stimulation. Consistent with these physiological findings, both groups demonstrated comparable clinical improvement at one-week follow-up, with no significant differences on either self-report scale.

Prior to conducting this study, our fundamental question was whether DCS could enhance aTMS effects as it does with daily or single-session protocols^4^, or whether high-intensity accelerated protocols already produce maximal plasticity that cannot be further enhanced (called occlusion) as has been established with strong LTP-inducing protocols^27, 28^. Our findings are consistent with the occlusion hypothesis: the 10-session, 1 800-pulse iTBS protocol delivered with 50-minute intersession intervals may saturate synaptic enhancement mechanisms, leaving no additional capacity for NMDAR mediated potentiation.

Very interestingly, and by contrast, a recent real-world clinical case series applied similar pharmacologic augmentation approach to 20 sessions of 600 pulses with 30 minutes between sessions and found robust and durable clinical benefits^16^. Current available data cannot fully explain the apparent difference between these outcomes, but if both are true, it is conceivable that 1 800 pulses, which was found to have the highest plasticity induction in the motor cortex^17^ compared to 600 and 1 200 iTBS pulses, may in fact be a stronger LTP-inducing protocol than 600 pulses which produces modest LTP in mouse hippocampal slices^29^. With this premise in mind, Vestring and colleagues demonstrated that strong LTP protocols cannot be enhanced by DCS, but weak ones can^28^. This is consistent with the daily TMS trials which paired DCS with 600 iTBS pulses producing large effects^3, 4^. Taken together, this highlights that parameter selection is especially sensitive to the brain state that NMDAR agonism introduces (Caulfield & Brown, 2022).

Our exploratory trial was focused on plasticity metrics and only assessed clinical responses at 1-week which was significantly improved in both groups, though the overall benefits were modest. By comparison, the case series by Vaughn et al. demonstrated an intermediate effect at 1-week, taking 1 month to reach maximal improvement, which was sustained at 3 months. However, it is difficult to compare the role of DCS between our findings since their clinical cases appropriately did not include a control for DCS. Given that we found no difference between groups, it is an empirical question whether 1800 pulses without DCS could produce the same effect. Other differences between these protocols which may justify future exploration include session number (10 vs 20), intertrain interval (27 vs 50 min), DCS dose (125 vs 250 mg) and timing (1 hour before TMS vs overnight) and an additional adjunct in some cases (a couple cases also received lisdexamfetamine). The lower response rates could also be a reflection of treatment resistance, as nearly half of our patients had previously received TMS with partial (reflecting those with mild baseline scores) or non-response, and 16% had ECT and 20% had ketamine. Finally, our placebo-controlled design could potentially account for a proportion of the apparent difference. Regardless, the retrospective clinical outcomes from Vaughn et al. (2025) are very promising and clearly justify future investigation.

If MEP assessments could serve as an index of plasticity induced by stimulation over the prefrontal cortex, including the dlPFC, this could be a useful response biomarker, as MEPs are very easy to obtain in the clinical setting. The dlPFC is both functionally^30^ and structurally^31^ connected to the M1 indirectly through the premotor region. Moreover, 10-Hz rTMS to the dlPFC has facilitated enhancement of MEPs^32^, which was also correlated with clinical improvement^33^, while 5- and 1-Hz decreased MEPs^34, 35^, although these findings have not been replicated across protocols like 600 pulse i/cTBS^36^ or rMT outcome measures^37^. Finally, the relationship between MEP plasticity before a clinical course of rTMS to the dlPFC and an improvement in depression was positively correlated in two studies^38, 39^. We sought to understand how accelerated TMS with DCS impacts distal cortical excitability and, to our knowledge, MEP changes throughout a course have not previously been reported on. Analysis of normalized MEP amplitudes revealed a significant interaction in pre-iTBS measurements, with PBO showing increasing trajectories and DCS remaining stable across sessions. However, post-iTBS measurements showed no group differences, indicating that DCS did not modulate immediate plasticity effects.

We administered 250 mg DCS the night before TMS, as opposed to our previous use of 100 mg, as a potential way to circumvent drug compounding access restrictions and to prevent challenges associated with some subjects having difficulty with the strict timing adherence which proved critical for optimal response^4^. We also thought a higher dose that likely remains in agonist range (< 250mg) would provide benefit since recent findings suggested that concentrations were strongly correlated with degree of clinical response^40^. However, higher DCS doses run a greater risk of NMDAR internalization induced by glycine receptor binding which is concentration-dependent^41^. Despite the higher dose, only one-third reached serum concentrations above 7 μg/mL, a concentration which has previously been associated with clinical remission in once daily treatment courses^40^. The reason for this is unclear, but laboratory assay inaccuracies are possible given that two participants demonstrated higher DCS levels at the end of the day and DCS takes 1–2 hours to peak in healthy volunteers^42^. Further limiting interpretation of this data, only 8 of 15 participants had both collection points due to difficult venipunctures.

This exploratory trial examined whether NMDAR agonism could enhance single-day accelerated iTBS through combined neurophysiological and clinical assessments. While the mechanistically-focused design was hypothesis-driven, outcomes were not preregistered as a clinical trial, and the reliance on self-report measures with brief follow-up limits clinical interpretation. We found no evidence that D-cycloserine modulated immediate plasticity responses to stimulation or enhanced clinical outcomes compared to placebo. The significant pre-iTBS interaction, reflecting differential corticospinal excitability trajectories between groups across the experimental day, suggests potential group differences in baseline stability or delayed facilitation effects, though the current design cannot distinguish these mechanisms. Both groups demonstrated modest clinical improvement from single-day accelerated iTBS, consistent with emerging evidence for ultra-condensed protocols^16^. These findings highlight the importance of parameter optimization in pharmacologically-augmented accelerated TMS and suggest that high-intensity protocols may saturate plasticity mechanisms differently than conventional approaches, warranting further investigation into optimal pulse numbers, intersession intervals, and drug timing for maximizing therapeutic benefit.

## Supplementary Material

Supplementary Files

This is a list of supplementary files associated with this preprint. Click to download.

- MAATsupp.docx

## Figures and Tables

**Table 1.** Demographic information. Error calculated as standard error of the mean.

Demographics	Total	PBO	DCS
N	30	15	15
Age	38.5 ± 16.0	41.8 ± 15.4	35.2 ± 16.4
Sex (n, % F)	18, 60.0%	11, 73.3%	7, 46.7 %
Race			
White (n, %)	29, 96.7%	15, 100%	14, 93.3%
Asian (n, %)	1, 3.3%	0, 0%	1, 6.67%
Prior neurotherapeutic trials	15, 50%	8, 53.3%	7, 46.7%
Prior TMS	14, 46.7%	7, 46.7%	7, 46.7%
Prior ECT	5, 16.7%	4, 26.7%	1, 6.7%
Prior Ketamine	6, 20%	3, 20%	3, 20%
**Psychotropic Medications**	19	9	10
Selective Serotonin Reuptake Inhibitor	12	6	6
Serotonin-Norepinephrine Reuptake Inhibitor	2	1	1
Atypical Antipsychotics	7	6	1
Stimulants	4	2	2
Benzodiazepines	3	2	1
Anti-epileptics	4	2	2
Norepinephrine-Dopamine Reuptake Inhibitors	5	2	3
Serotonin Antagonist and Reuptake Inhibitor	3	0	3
Z-drugs	2	1	1
Noradrenergic and Specific Serotonergic Antidepressants	2	0	2
**Clinical Severity**			
Baseline QIDS-SR16	14.0 ± 0.846	14.9 ± 1.30	13.1 ± 1.07
Final QIDS-SR16	11.4 ± .976	11.9 ± 1.64	10.9 ± 1.10
Percent Improvement QIDS-SR16 (%)	16.4 ± 5.6%	22.2 ± 7.07%	10.5 ± 12.5%
Baseline PHQ-9	14.7 ± 1.0	15.9 ± 1.4	13.3 ± 1.3
Final PHQ-9	12.7 ± 1.0	13.5 ± 1.7	11.8 ± 0.9
Percent Improvement PHQ-9 (%)	9.6 ± 6.9%	16.4 ± 6.1%	2.3 ± 12.7%
**Clinical Outcomes**			
QIDS-SR16 Response	20.0%	20.0%	20.0%
QIDS-SR16 Remission	16.7%	20.0%	13.3%
PHQ-9 Response	10.3%	6.7%	14.3%
PHQ-9 Remission	6.9%	6.7%	7.1%
**Motor-Evoked Potentials**			
Baseline MEPs (Pre-Tx 1) (mV)	1.06 ± 0.17	0.79 ± 0.21	1.34 ± 0.25
